# Motion based ex vivo (MOTEX) culture of breast tumor slices sustains microenvironment composition

**DOI:** 10.1016/j.neo.2025.101221

**Published:** 2025-08-21

**Authors:** Zofia M. Komar, Mieke Bavelaar, Ellen Kageler, Nicole S. Verkaik, Mandy M. van Rosmalen, Carolien H.M. van Deurzen, Michael A. den Bakker, Roland Kanaar, Adriaan B Houtsmuller, Thierry P.P. van den Bosch, Agnes Jager, Dik C. van Gent

**Affiliations:** a Department of Molecular Genetics, Erasmus MC Cancer Institute, Rotterdam, The Netherlands; b Department of Medical Oncology, Erasmus MC Cancer Institute, Rotterdam, The Netherlands; c Department of Pathology, Erasmus MC, Rotterdam, the Netherlands; d Department of Pathology, Maasstad Ziekenhuis, Rotterdam, the Netherlands; e Erasmus Optical Imaging Center and Department of Pathology, Erasmus MC, Rotterdam, the Netherlands; f Oncode Institute, Erasmus MC, Rotterdam, The Netherlands

**Keywords:** Tumor microenvironment, Breast cancer, Treatment prediction, Tumor infiltrating lymphocytes (TILs)

## Abstract

Personalized medicine for breast cancer (BrC) requires predictive biomarkers to select the optimal therapeutic option for each individual patient. Personalization of chemotherapy or immunotherapy responses is particularly challenging, as molecular markers do not appear to be sufficiently predictive for therapy response. Functional assays for therapy selection may be the solution for this dilemma. An interesting approach is *ex vivo* cultures of precision cut tumor slices, such as the MOtion-based Tissue EX vivo (MOTEX) method that we described previously. This culture method has the advantage that it carries all cell types in the tumor, including various immune cell populations. We here show, that macrophages, B-cells and T-cell populations are maintained in the MOTEX culture for several days without apparent loss of viability. Even treatment with the microtubule poison paclitaxel did not reduce immune cell abundance or viability significantly. Anthracycline-based chemotherapy, however, did affect immune cell composition, as expected based on its cytotoxic properties. Therefore, we conclude that MOTEX culture of BrC tissue slices can be used to investigate effect of treatments that involve the immune system. This opens perspectives to develop predictive assays for immune checkpoint inhibitor treatment and other therapeutic interventions that require immune components in the assay system.

## Introduction

Breast cancer (BrC) represents a substantial global health burden, with one in 20 women worldwide expected to be diagnosed with BrC in their lifetime [1]. This risk is even higher in Western countries; for example, approximately one in seven women in the Netherlands will develop BrC during their lifetime [2]. As a result of extensive efforts to improve the early detection and treatment efficacy, the survival rates of BrC patients have increased over the recent years [3]. With the advancements in treatment options available for BrC patients, the choice of the most appropriate therapy for individual patients became a challenge. To overcome this, functional *ex vivo* sensitivity tests have been developed to enable prediction of the treatment outcome in patients [[4], [5], [6], [7]]. An example of such a culture method, previously developed by our group [8], uses tissue slice cultures referred to as MOtion-based Tissue EX vivo (MOTEX). This method allows preservation of tumor cell viability for up to seven days, during which the chemotherapy treatment *ex vivo* can be applied. The MOTEX culture has successfully been implemented in development of prediction tests for cisplatin [9], microtubule inhibitors [9,10] and anthracyclines [11]. Investigating tumor cell responses resulted in a concordance of 75 % between MOTEX and *in vivo* responses in a clinical proof-of-concept study for anthracycline-sensitivity [11]. Although this result is promising, it will be important to further increase the predictive power of the assay by adding additional analysis methods.

The tumor microenvironment (TME) is an important factor in cancer treatment outcome and prognosis [12]. It is a complex network of interactions between cancer cells and the non-malignant stroma containing fibroblasts, blood vessels, extracellular matrix and immune cells [13,14], which plays an important role in chemotherapy resistance [15,16] and treatment response [17] in BrC patients. Considering that a major advantage of a tissue slice culture (such as MOTEX), is the presence of the intact tissue architecture and TME, it is essential to determine whether the components of the TME are present and viable in the system before and after the *ex vivo* culture and how they react to the treatment *ex vivo.*

The aim of this study was to investigate the preservation of the TME in MOTEX culture, and the effect of *ex vivo* treatment with taxanes (paclitaxel and docetaxel) and anthracycline-containing (FAC) chemotherapy on the TME composition.

## Materials and methods

### Material collection, handling and processing

In this study, primary BrC tissue was tested for taxane sensitivity *ex vivo* and for anthracycline sensitivity both *ex vivo* and *in vivo* (Table 1).

**Table 1:** Overview of the primary breast cancer material used in this study.

Tumor type	Method	Sensitivity ex vivo	Sensitivity in vivo	Material collection
Primary breast cancer	**(A) Resection** material	Taxanes (the REMIT assay) [10]	*Not tested*	Most samples collected for this study. Part previously used [10].
Primary breast cancer	**(B) Biopsy** material (BREAST study)	FAC (EdU, TUNEL and H&E) [11]	Anthracycline-based chemotherapy (BREAST study)	All of the samples were collected previously [11].

Resection material (A) was prospectively collected from patients undergoing wide local excision or ablation at the Erasmus MC Cancer Institute or Maasstad Hospital in Rotterdam, The Netherlands. Following the macroscopic evaluation of the surgical specimens by pathologists, fresh residual tumor tissue was collected for research purposes in compliance with the Code of Proper Secondary Use of Human Tissue in the Netherlands, established by the Dutch Federation of Medical Scientific Societies. This was conducted in accordance with the ethical principles outlined in the Declaration of Helsinki. The ethical committee has waived the need for written informed consent, and patients who objected to the secondary use of residual tumor material for research were excluded from the study. The Erasmus MC Medical Ethics Commission (MEC-11-098) approved this protocol.

Following surgery, the resection material was collected in customized breast medium, containing: phenol-free DMEM:HAM F12 (1:1) and DMEM, mixed in 2:1 ratio respectively; fetal bovine serum (2 %; FBS; Bondico BV, Alkmaar, The Netherlands), hydrocortisone (0.3 µg/ml, Sigma-Aldrich, St. Louis, MO, USA), insulin (4 µg/ml, Sigma-Aldrich, St. Louis, MO, USA), transferrin (4 µg/ml; Sigma-Aldrich, St. Louis, MO, USA), 3,3´,5 Triiodothyronine (1.3 ng/ml, Sigma-Aldrich, St. Louis, MO, USA), epidermal growth factor (EGF; 8 ng/ml; Sigma-Aldrich, St. Louis, MO, USA)), choleratoxin (7 ng/ml, Sigma-Aldrich, St. Louis, MO, USA), adenine (0.2 mg/ml, Sigma-Aldrich, St. Louis, MO, USA) and penicillin streptomycin (1 %; Sigma-Aldrich, St. Louis, MO, USA); and processed as described previously [8,9]). In short, samples were cut into 300 μm thick slices using a Leica VT 1200S Vibratome (Leica Microsystems, Wetzlar, Germany) and cultured in breast medium at 37°C in a 5 % CO_2_ humidified incubator with continuous rotation (60 rpm) provided by a Stuart SSM1 mini orbital shaker (Camlab Ltd, Cambridge, UK), referred to as MOTEX culture. During the culture period, tissue slices were treated for three days with docetaxel (Biovision, ITK Diagnostics, Uithoorn, The Netherlands) or paclitaxel (Sigma-Aldrich, St. Louis, MO, USA). To perform the REplication MITosis (REMIT) assay for taxane sensitivity [9,10], 30 μM 5-Ethynyl deoxyuridine (EdU) (Invitrogen, Carlsbad, CA, USA) was added to the culture medium 2 hours before fixation. Finally, tissue slices were fixed in 10% neutral buffered formalin for 24-72 hours at room temperature, embedded in paraffin and sectioned. To ensure that the majority of the tissue slice is present in the microscopic section and correctly oriented, each slice was carefully flattened and positioned at the bottom of the mold during paraffin embedding.

Primary BrC biopsy material (B) was originally collected in the BREAST study [11]. This study was performed in line with the principles of the Declaration of Helsinki. The Ethics Committee of the Erasmus MC (MEC 16-600 / Netherlands Trial register NL5588) granted approval. In short, patients included in the study had already been scheduled to start neo-adjuvant anthracycline-containing chemotherapy. After informed consent was signed, two fresh pretreatment biopsies of the breast tumor were collected and further processed by the pathology department or the research lab. The goal of the study was to validate the anthracycline sensitivity prediction assay in a proof-of-concept study [11]. The residual material of both biopsies remaining after finalization of the BREAST study has been used in this research project.

Assessment of both the *ex vivo* and *in vivo* sensitivity to FAC (combination of 5-fluorouracil, doxorubicin and 4-hydroperoxy cyclophosphamide) and anthracycline-containing chemotherapy was described previously [11]. In short, tissue slices were treated with FAC. Subsequently, the TUNEL/DAPI, EdU/DAPI and H&E were used to assess FAC sensitivity *ex vivo*. Patient sensitivity to the treatment *in vivo* was based on the MRI response after four courses of anthracycline-containing chemotherapy. Patients were considered sensitive to the treatment if >50% decrease in tumor size was observed and intermediate if <50% decrease and <20% increase in tumor size was registered. No patients included in the BREAST study showed resistance to the treatment.

### Staining and microscopic analysis

Staining was performed to determine levels of apoptosis (TUNEL), taxane sensitivity (the REMIT assay), morphology (H&E), general immune levels (Immunohistochemistry (IHC) for CD45) and specific cell types (Multiplex Immunofluorescence (MIF) for pan-cytokeratin (PCK), CD20, CD4, CD8, CD68, CD163) (Table 2).

**Table 2:** Overview of the stainings performed in this study.

Sample type	TUNEL	H&E	IHC (CD45)	MIF Panel 1 (PCK, CD20, CD8, CD4 and CD68)	MIF Panel 2 (PCK, CD4, CD8, CD68 and CD163)
(A) Resection material	Supplementary Fig. 1	General morphology assessment	Fig. 1, 2, 3	Fig. 1	Fig. 2,3
(B) Biopsy material (BREAST study)	**-**	General morphology assessment	Fig. 1, 2, 4,5	Fig. 1	Fig. 2, Fig. 4, Fig. 5

### Immunohistochemistry (IHC) and immunofluorescence

Histological tumor architecture was examined using H&E staining. Additionally, CD45 immunohistochemical staining was performed with an automated, validated and accredited staining system (Ventana Benchmark ULTRA, Ventana Medical Systems, Tucson, AZ, USA) using ultraview or optiview universal DAB detection Kit (Ventana). Following deparaffinization and heat-induced antigen retrieval, the tissue samples were incubated according to their optimized time with the CD45 clone 30-F11 antibody (0.5 mg/ml; 103102, Biolegend, San Diego, CA, USA). The incubation was followed by hematoxylin II counter stain for 12 minutes and then a blue coloring reagent for 8 minutes. The immunohistochemistry (IHC) slides were imaged using a NanoZoomer Digital Pathology-HT (C9600-03) slide scanner with Fluorescence Illumination Optics (L10387) (Hamamatsu Photonics, K.K., Japan).

The terminal deoxynucleotidyl transferase dUTP nick end labelling (TUNEL) assay was performed as described previously [8]. *Ex vivo* taxane sensitivity was previously determined for most samples and carried out for the remaining samples, as described previously [9,10].

### Multiplex immunofluorescence (MIF)

The assessment of immune cell populations within the TME was conducted through 5-plex immunofluorescent staining (previously developed by PARTS - Department of Pathology, Erasmus MC [18]), allowing for the simultaneous detection of multiple immune markers (Table 3). The staining was conducted using an automated staining system, the Ventana Benchmark Discovery (Ventana Medical Systems, Tucson, AZ, USA). First, the antigen retrieval was performed by applying heat and pressure in combination with a suitable retrieval buffer. Next, the tissue sections were incubated, in parallel, with a panel of primary antibodies conjugated to a unique fluorophore. The Tyramide Signal Amplification antibody stripping protocol was used to remove antibodies from the sample and minimize the risk of cross-reactivity. Finally, the tissue was stained with DAPI, mounted with Vectashield mounting medium (Vector Laboratories Cat. No. H-1000) and imaged using the Zeiss Axio Imager II fluorescence microscope.

**Table 3:** List of antibodies used for multiplex immunofluorescence.

Antibody	Catalogue No.	Company	Clone	Concentration
PCK	NBP2-29429	Novus Biologicals	AE-1/AE-3	0.2 mg/ml
CD4	NBP1-19371	Novus Biologicals	Polyclonal	1.0 mg/ml
CD8	sc-7188	Santa Cruz	D-9	0.2 mg/ml
CD68	sc-7084	Santa Cruz	M-20	0.2 mg/ml
CD20	NBP2-44745	Novus Biologicals	IGEL/773	0.2 mg/ml
CD163	05973929001	Cell Marque	MRQ-26	0.2 µg/ml
CD80	ab134120	Abcam	EPR1157(2)	20 µg/ml

### Image acquisition and analysis

IHC and MIF stainings were analyzed using QuPath software [19]. First, the image type was chosen (IHC or MIF) and regions of interest (ROIs) were manually assigned. The positive cells detection tool was used with thresholds kept equal for all IHC stainings analyzed in this project. Two types of MIF staining were carried out: panel 1 consisting of PCK, CD4, CD8, CD68 and CD20; and panel 2 consisting of PCK, CD4, CD8, CD68 and CD163. The first panel allowed assessment of the presence of T cells, macrophages, and B cells within the tested samples. To better understand the response of the TME to the treatment *ex vivo*, this panel was modified by replacing the B-cell marker (CD20) with a marker specific for M2 macrophages (CD163), enabling discrimination between M1 and M2 macrophage populations. For both panels, the cell detection tool was used to annotate cells based on the DAPI signal. Due to the differences in the expected overlap of the used markers (in panel 2 CD163 is expected to overlap with CD68), different training methods were used for quantification of these stainings. For MIF panel 1, all markers were trained simultaneously using the artificial neural network (ANN_MLP) classifier. Most markers were trained based on all of the available features, except for PCK (cytoplasmic marker) which was trained only based on the cytoplasmic signal. Samples stained with MIF panel 1 were subsequently analyzed by loading the classifier on a merged image containing all markers. For MIF panel 2, markers were trained individually or in groups. Due to a significant overlap of PCK in the CD4 channel, the CD4 classifier was trained together with the PCK signal – cells that contained only CD4 signal were considered positive and cells with both CD4 and PCK as negative. The classifier for CD163 was trained together with the CD68 marker – only cells showing both markers were considered as CD163-positive. Remaining markers were trained separately. After the training, all markers were analyzed separately.

To quantify levels of apoptosis, microscopic images were acquired using the Automated Upright Microscope Leica DM4000 B with a magnification of 200x (Leica Microsystems, Wetzlar, Germany). If possible, ten fields of view were imaged per condition. For small sample sizes, a minimum of six fields of view were acquired. Images were subsequently analyzed using the ImageJ software on the basis of a total number of pixels from the green channel (TUNEL) and the blue channel (DAPI). Next, the percentage of the TUNEL signal was calculated out of the total of the DAPI signal, as described previously [10].

## Results

### MOTEX culture preserves the leukocytes within BrC tissue slices

**Fig. 1:**
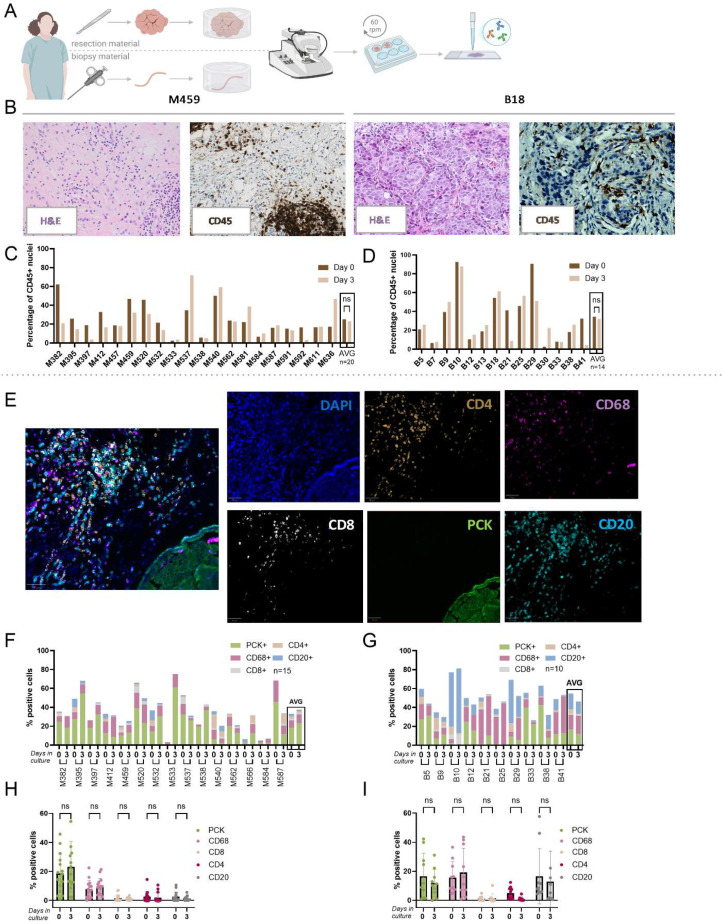
*Maintenance of TME in MOTEX culture for 3 days.* (A) Schematic overview of tissue collection and handling. Tumor specimens were embedded in agarose and sliced using a Leica Vibratome. Next, generated slices were cultured in the MOTEX system for three days, with or without treatment. Formalin-fixed paraffin-embedded (FFPE) samples were then sectioned, stained and imaged. (B) Representative images of the H&E and CD45 staining of two primary BrC resection and two biopsy samples. (C) CD45 levels of the resection and (D) biopsy samples at day 0 and day 3 of culture (n=20 and 14, respectively). (E) Representative images of the Panel 1 MIF of resection BC material (sample M395). (F) Quantification results of markers representing the TME of the resection and (G) biopsy material at day 0 and day 3 of culture. The Y-axis indicates the percentage of cells positive for the indicated surface marker in the tissue slice. (H) Quantification results from Fig. F, with differences between all markers plotted separately. Significant differences are indicated in the graph based on the 2-way ANOVA test. (I) Quantification results from Fig. G, with differences between all markers plotted separately. Significant differences are indicated in the graph based on the 2-way ANOVA test.

The TME plays a crucial role in mediating tumor responses to therapeutic interventions. Therefore, composition and survival of the TME under MOTEX culture was investigated in tissue slices generated from primary BrC resection and biopsy material (Fig. 1). First, the tissue morphology was assessed by H&E staining, to ensure that only samples containing tumor cells were used for further analysis (Fig. 1B). Next, CD45 staining [20] was performed to assess the percentage of leukocytes within the slice before and after three days of culture (Fig. 1B-D). Generally, the leukocyte levels remained unchanged after MOTEX culture. Expectedly, high heterogeneity was observed between the different primary BrCs. The 5-plex staining showed that all tested cell types - tumor cells (Pan-Cytokeratin; PCK), macrophages (CD68), B-cells (CD20) and cytotoxic T (CD8) and T-helper (CD4) cells - were present in the tested samples (Fig. 1E). Overall, all cell types were maintained in the tissue slices after three days of culture (Fig. 1F-G) and no induction of apoptosis was observed (n=13; Supplementary Fig. 1A). Some resection material samples were cultured longer (five and/or seven days), showing no decline in the CD45⁺ levels and presence of all cell types at the later time points (Supplementary Fig. 1B-D). We conclude that all cell types are still viable at three days of MOTEX culture (the time point used for most sensitivity read-outs [[9], [10], [11]]), as they were still detected after five to seven days of culture.

### Evaluation of the heterogeneity within the primary BrC samples

**Fig. 2:**
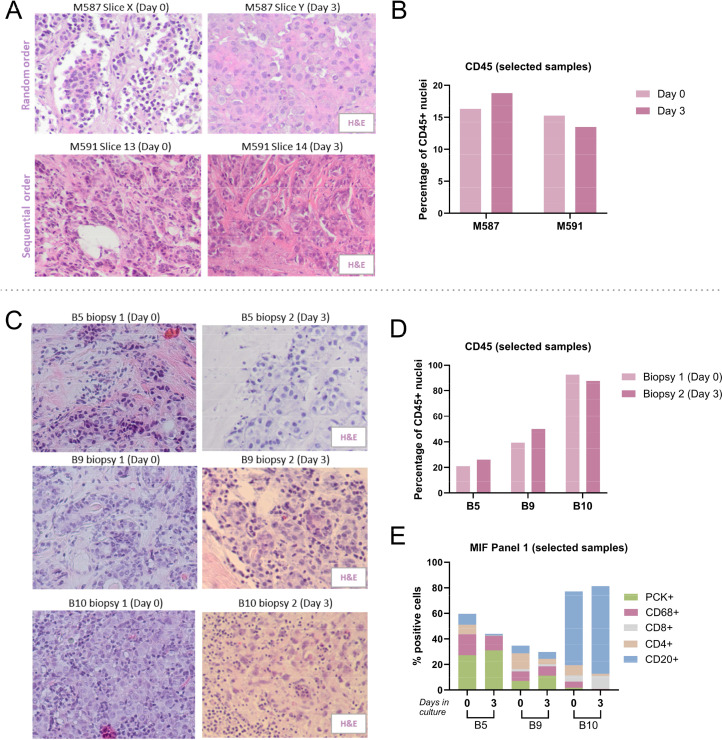
*Similarities between tissue slices in primary breast cancer specimens.* (A) H&E images of two primary tumor resection samples, where slices were assigned randomly (generated slices were mixed during slicing and randomly assigned to each condition; M587) and in a sequential order (the order of slicing was kept when assigning each condition; M591). (B) CD45 levels for these two samples. (C) H&E images of three primary tumor biopsy samples (B5, B9 and B10), showing differences in morphology between two biopsies – one fixed immediately after biopsy collection and the other one cultured for three days. (D) Levels of CD45⁺ cells and (E) Panel 1 MIF of the three selected samples. The Y-axis indicates the percentage of cells positive for the indicated surface marker in the tissue slice.

One of the main challenges of using a tissue slice culture system to assess treatment outcome is the high heterogeneity present within the tumor and between the tissue slices [21]. Therefore, we have evaluated the heterogeneity present in the sample set used in this study and its potential effect on the accuracy of the data. Significant differences were observed between the different samples, but also within different tissue slices originating from the same patient. Most tissue slices were randomly distributed between conditions following the slicing (M382-M587), which resulted in a relatively high level of heterogeneity in the morphology of the slices. For this reason, several samples collected afterwards (M591-M636), were distributed in sequential order, resulting in a more comparable morphology of the tissue slices (Fig. 2A). However, in spite of the differences in morphology, both samples M587 and M591 showed comparable CD45⁺ levels, regardless of the sequence of the tissue slices used (Fig. 2B), suggesting that immune cell infiltration might be relatively constant throughout the tumor.

To determine the level of heterogeneity in the samples used in this study, we compared the two different biopsies collected from the same patient for the BREAST study sample set (biopsy material). These samples did not only represent two different regions of the tumor, but also different time points at which they were fixed – immediately after the procedure and after three days in culture. Interestingly, similarities in the morphology (H&E staining) were observed between the two different biopsies (Fig. 2C). Moreover, general levels of immune cells (CD45⁺ cells) were also comparable between most samples tested (Fig. 1, Fig. 2D). A more precise analysis of the different cell types in the TME (Panel 1 MIF) revealed slight differences between the different samples, but general trends of the cell composition remained comparable (Fig. 2E). These results suggest that the heterogeneity observed within the tumor and between the tissue slices has a relatively limited effect on the read-outs applied in this study.

### TME composition after ex vivo chemotherapy treatment

**Fig. 3:**
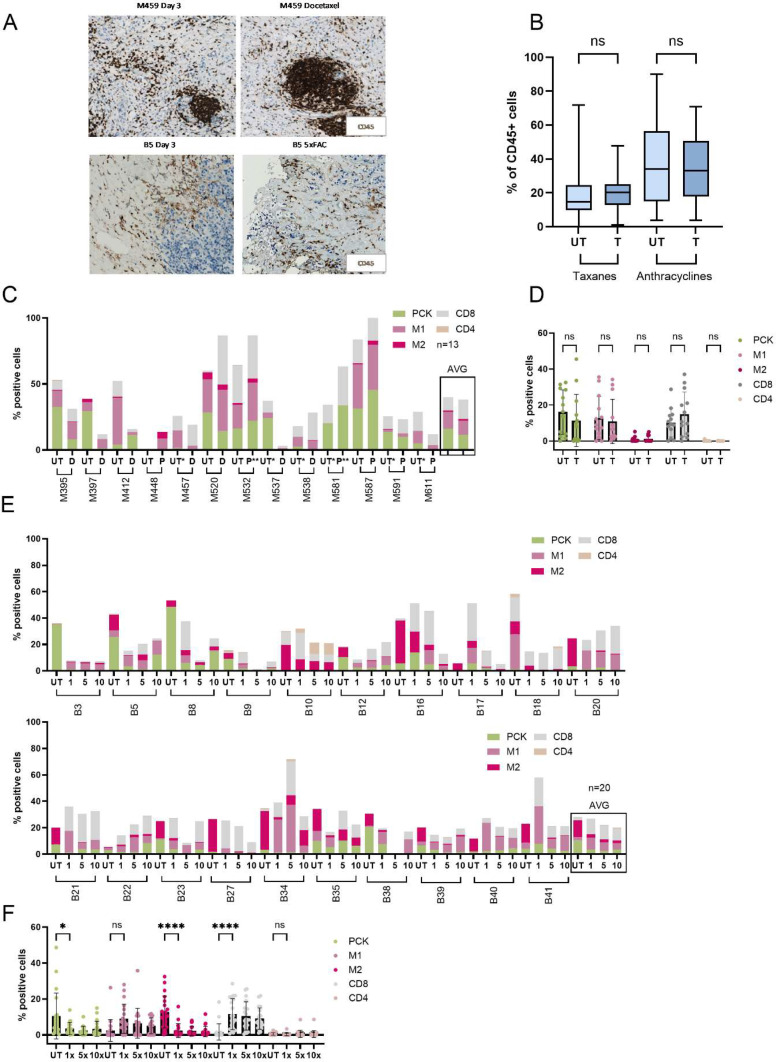
*Changes in the TME after treatment ex vivo.* (A) Representative images of the CD45 DAB staining of M459 (resection) and B5 (biopsy) BC samples before and after treatment. (B) Quantification results of the CD45 staining before and after taxane and FAC treatment. For taxanes, a concentration of 10 nM docetaxel or paclitaxel was used, while for the FAC the average was calculated after treatment with all three different treatment concentrations (FAC 1x, 5x and 10x). Significant differences are indicated in the graph based on the 2-way ANOVA test. (C) Quantified results of the panel 2 MIF staining for the resection material samples without and with paclitaxel (P) or docetaxel (D) treatment. The Y-axis indicates the percentage of cells positive for the indicated surface marker in the tissue slice. In most samples, day 3 was used as a reference point and 10 nM taxane as treated sample. In some samples, due to lack of material, Day 0 or 5 was used as an untreated sample and 25 nM taxane treatment as treated samples (indicated with a “*” for days in culture or “**” for higher treatment concentration). AVG = average of all samples combined. (D) Quantification results from Fig. C, with differences between all markers plotted separately. Significant differences are indicated in the graph based on the 2-way ANOVA test. (E) Quantified results of the panel 2 MIF staining for the biopsy samples without and with FAC (indicated as T for treatment, calculated as average response to all tested FAC concentrations). The Y-axis indicates the percentage of cells positive for the indicated surface marker in the tissue slice. (F) Quantification of results from Fig. E, with differences between all markers plotted separately. Significant differences are indicated in the graph based on the 2-way ANOVA test.

Treatment induced changes of the TME composition may influence treatment efficacy. Therefore, we analyzed the levels of immune cells before and after *ex vivo* treatment. We found that taxane (docetaxel or paclitaxel; resection material) and FAC treatment (biopsy material) did not affect general immune cell levels (Fig. 3A,B and Supplementary Fig. 2). More detailed analysis of TME composition did not show major differences in immune composition after *ex vivo* taxane treatment (Fig. 3C,D). However, some differences were observed after *ex vivo* FAC treatment (FAC; biopsy material) (Fig. 3E,F). More specifically, a significant decline was shown for tumor cells (PCK) and M2 macrophages (confirmed by an increase in M1 macrophages in Supplementary Fig. 2E), while a significant increase was shown in the CD8⁺ T-cell population (Fig. 3F). The increase in CD8⁺ cells is likely linked to the decline in tumor cells (PCK), resulting in a change in the composition of the tumor slice. Additionally, the decline in M2 macrophages may indicate a transition toward a more pro-inflammatory anti-tumor response upon FAC treatment [22]. Additional assessment was performed to determine whether differences in the TME response to treatment were present between BrC subtypes (Supplementary Fig. 2F and G). We found that FAC treatment induced more extensive changes in the TNBC tumors (n=7). However, both subtypes showed the same trends in immune cell types in ER positive BrC (Fig. 3G).

### Increased pretreatment T-cell levels in *ex vivo* taxane sensitive samples

**Fig. 4:**
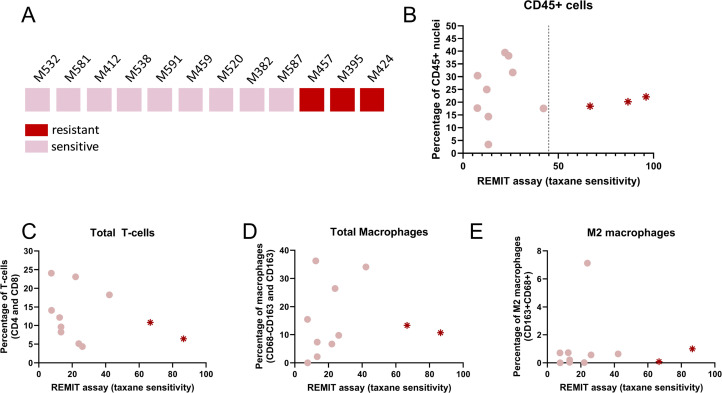
*Assessment of the initial levels and composition of the TME and taxane response ex vivo.* (A) Schematic overview representing resection samples with known taxane sensitivity, scored as either sensitive or resistant to the treatment. The sensitivity to taxanes is based on the EdU/pH3 ratio after 10nM of taxane treatment relative to the untreated sample. Graphs B-E show division of the samples on the X-axis, based on their REMIT score. Samples M457 and M520 were assessed within this study, scoring as resistant (67 %) and sensitive (24 %), respectively. (B-H) Scatter plots representing the CD45⁺ (B), total T-cell (C), total macrophages (D) and the M2 macrophage (E) levels in relation to the taxane sensitivity. Results from graphs C-E are based on the panel 2 MIF results represented in Fig. 1, Fig. 2. All graphs include the average levels calculated from day 0 and day 3 of untreated samples; exceptions are samples M412 and M587 were only day 3 was used and M457, M538, M581 and M591 were only day 0 was used (due to insufficient availability of material for the other time points). Panel 2 MIF results from sample M424 were not available, and this sample was only included in Fig. 3B.

Subsequently, we asked whether analysis of the TME could contribute to our recently developed taxane sensitivity assay [9,10]. We investigated the initial composition of the TME in relation to the taxane response *ex vivo*. Nine taxane sensitive and three resistant samples were analyzed (Fig. 4A and Supplementary Table 1). We found that the five samples with highest CD45⁺ levels (M459, M520, M382, M581 and M412), were all classified as taxane sensitive *ex vivo*. However, the remaining four taxane-sensitive samples showed relatively lower CD45⁺ levels (Fig. 4B). Next, the more detailed composition of the TME was considered by quantifying T-cells and macrophages. Although samples with the highest total T-cell levels were generally taxane sensitive, low levels of T-cells were found in both sensitive and resistant samples (Fig. 4C). The five samples that showed increased T-cell levels (M532, M459, M587, M581, M412) were all scored as taxane sensitive. Additionally, the ratio of CD4⁺/CD8⁺ levels, the relative decline of T-cells, total macrophage levels, the M1/M2 ratio and the levels of M2 macrophages were investigated (Fig. 4 and Supplementary Fig. 3) with none of these markers showing a clear trend that could be correlated with taxane sensitivity *ex vivo*. In conclusion, we found a trend for taxane sensitive samples towards higher CD45⁺ and T-cell levels in pretreated samples, but no clear correlation was found between the TME levels and taxane sensitivity *ex vivo*. We conclude that the functional sensitivity and TME composition are two independent parameters. The possible improvement of the predictive power of *ex vivo* assays using TME analysis needs to be evaluated in a larger cohort study.

### TME and FAC response

**Fig. 5:**
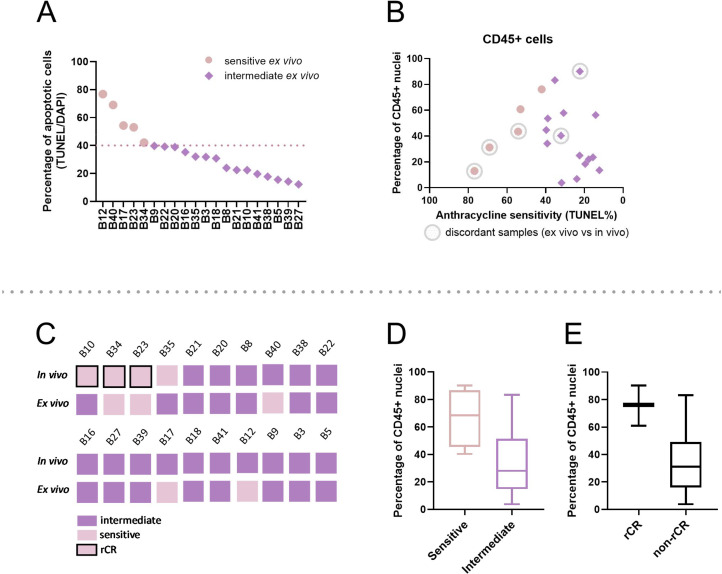
*Assessment of the pretreatment TME composition and sensitivity to FAC ex vivo and anthracycline-based chemotherapy in vivo.* (A) Representation of the TUNEL levels measured in the BREAST study [11] after treatment with 1xFAC, used to categorize the biopsy samples in a gradient from the least to most sensitive tumors *ex vivo*. (B) Scatter plot representing the CD45⁺ levels in relation to FAC sensitivity *ex vivo*. (C) Schematic overview of the *ex vivo* and *in vivo* sensitivity to anthracycline-based chemotherapy of biopsy samples, scored as sensitive or intermediate to the treatment. Patients with rCR were marked accordingly. (D, E) Average CD45⁺ levels divided between sensitive and intermediate and rCR and non-rCR patients.

Subsequently, we tested how the initial immune levels correlate with FAC response *ex vivo*. Five sensitive biopsy samples and 15 samples with intermediate sensitivity from the BREAST study [11] were analyzed (Fig. 5). We divided the samples in a gradient from the most to the least sensitive on the basis of apoptosis levels after *ex vivo* treatment with 1xFAC (Fig. 5A). The initial levels of immune cells did not show a clear correlation with the *ex vivo* FAC sensitivity (Fig. 5B). However, samples showing *ex vivo* sensitive and *in vivo* intermediate phenotype all had relatively low immune infiltration, which may have contributed to this difference. Total T-cell levels before FAC treatment did not show a clear correlation with the *ex vivo* sensitivity (Supplementary Fig. 4A). Finally, the total levels of macrophages and total levels of M2 macrophages before treatment were determined (Supplementary Fig. 4B,C). Generally, higher levels of M2 macrophages were found in samples that were less sensitive to FAC treatment. To further explore these results, we extended the analysis to investigate whether pretreatment immune cell levels could add to the prediction of FAC treatment sensitivity *in vivo*. Samples were categorized based on their sensitivity measured *in vivo* (MRI response) with four samples showing sensitivity - from which three a radiological complete response (rCR) – and 16 intermediate response to anthracycline-based treatment (Fig. 5C). Initial levels of immune cells (CD45⁺) were increased in patients sensitive to anthracycline-based treatment, with samples of the most sensitive patients - B10 (ER+, HER2-), B34 (ER-, HER2-) and B23 (ER-, HER2-) (Supplementary Table 2) - showing the highest numbers of CD45⁺ cells. Nevertheless, high immune infiltration was also found in one intermediate sample, B16 (ER+, HER2+). On average, a slight difference was observed between the sensitive and intermediate models (Fig. 5D), which became more pronounced when the division was made between the rCR and non-rCR patients (Fig. 5E). The remaining analyses of the T-cell and macrophage levels did not show significant differences between the sensitive and intermediate patients (Supplementary Fig. 4). Surprisingly, the M2 macrophage levels showed an opposite trend when compared to the *ex vivo* sensitivity to FAC (Supplementary Fig. 4G). In conclusion, the composition of the TME does not seem to correlate well with the known FAC sensitivity *ex vivo*. Increased immune infiltration (CD45⁺ cells) was found in the most sensitive patients, suggesting that a combination of this read-out with the functional assay could improve the predictive value.

## Discussion

We show that the MOTEX culture preserved the TME in the primary BrC tissue slices. The TME composition showed no clear differences after taxane treatment and only slight changes after FAC treatment. The assessment of heterogeneity in the material used in this study revealed a limited degree of variation, which did not appear to influence the cell composition of the TME.

One of the advantages of the tissue slice culture is the preservation of the TME. Nevertheless, for many years this claim was made solely based on the tumor morphology assessment (e.g. H&E), without detailed analysis of separate components of the TME [5,6,8]. In this study, we demonstrate that the previously developed MOTEX culture indeed maintains the survival of the TME for several days, showing great potential of this method for various chemotherapy prediction assays. Moreover, it is an interesting model for immune checkpoint inhibitor evaluation. As immunotherapy for BrC treatment shows less effectivity than initially expected [23,24], there is a growing demand for models that can predict and help optimize treatment outcome. The main disadvantage of using the MOTEX culture system for both immunotherapy and chemotherapy testing in its current form is the absence of circulating immune cells. However, this could be addressed by introducing autologous immune cells to the cultures [25]. We consider that the lack of circulating immune cells might cause depletion of the resident immune cells in the tissue slice. However, we found no differences in the TME composition following taxane treatment, while only subtle differences in the TME composition were observed after FAC treatment, most likely due to tumor cell death and subsequent shift in relative cell composition.

To further assess the suitability of this assay for clinical application, we considered the degree of heterogeneity in the group of tested primary BrC tissue slices. First, two different types of primary material were used - resection or biopsy – showing no significant differences between them. Secondly, the two separate biopsy samples originating from the BREAST study [11] showed limited differences in immune cell composition. Considering that some heterogeneity was still observed between the tissue slices used for the read-outs, we confirmed that collection of consecutive slices can help reduce heterogeneity between slices that are used for assessing treatment outcome. These results show that general heterogeneity associated with tissue slice culture is manageable using slight adaptations to the experimental setup. This approach should now be validated in a larger cohort study, where conclusions can be drawn on the correlation with tumor response in patients.

To evaluate the potential of the pretreatment TME assessment as additional read-out that could improve the previously developed sensitivity prediction assays [[9], [10], [11]], we examined whether any trends could be observed between TME levels and treatment response to taxanes *ex vivo*, as well as to FAC both *ex vivo* and *in vivo.* In general, the most taxane sensitive samples showed relatively high T-cell levels. This is in line with a recent study, showing that paclitaxel-induced T-cell activation is essential to achieve tumor killing in the tested model [26]. Nevertheless, limited data is available linking pretreatment TME composition to taxane monotherapy response in patients, further highlighting the importance of performing such assessments in a larger cohort. Most studies compare TIL levels with the response to neoadjuvant chemotherapy, usually based on a combinatory treatment with both taxanes and anthracyclines [27,28]. These studies show that high TIL infiltration is mostly linked with better prognosis. In our study, the pretreatment TME composition was also compared with the *ex vivo* FAC and *in vivo* anthracycline-based chemotherapy treatments. We found that high levels of CD45⁺ cells were found in most *in vivo* sensitive samples, consistent with other studies showing that increased TIL levels are generally associated with better response to anthracycline-based chemotherapy [29,30]. Moreover, high levels of M2 macrophages were found in the *ex vivo* intermediate samples, which can also be supported by multiple studies linking high M2 macrophage levels with poorer prognosis [[31], [32], [33]].

In conclusion, this study demonstrates that the TME is preserved in tissue slices during MOTEX culture, which provides a model that closely reflects the *in vivo* tumor composition. In general, we did not find a strong correlation between the pretreatment TME composition and sensitivity to taxanes or FAC treatment *ex vivo*. This suggests that both read-outs are independent from each other and could potentially be used in a complementary manner. This should now be validated in a larger cohort of samples, to determine whether the addition of TME analysis to *ex vivo* sensitivity measurements can improve the predictive value of these tests. Considering that these assays can be performed on the material remaining after the functional test has been performed, it can be easily implemented in future research projects.

## CRediT authorship contribution statement

**Zofia M. Komar:** Writing – original draft, Visualization, Project administration, Methodology, Investigation, Data curation, Conceptualization. **Mieke Bavelaar:** Writing – review & editing, Investigation, Data curation. **Ellen Kageler:** Writing – review & editing, Investigation. **Nicole S. Verkaik:** Writing – review & editing, Investigation. **Mandy M. van Rosmalen:** Writing – review & editing, Resources, Data curation. **Carolien H.M. van Deurzen:** Writing – review & editing, Resources, Methodology. **Michael A. den Bakker:** Writing – review & editing, Resources. **Roland Kanaar:** Writing – review & editing, Supervision, Funding acquisition. **Adriaan B Houtsmuller:** Writing – review & editing, Supervision, Funding acquisition. **Thierry P.P. van den Bosch:** Writing – review & editing, Resources, Methodology. **Agnes Jager:** Writing – review & editing, Writing – original draft, Supervision, Funding acquisition, Conceptualization. **Dik C. van Gent:** Writing – review & editing, Writing – original draft, Conceptualization.

## Declaration of competing interest

The authors declare that they have no known competing financial interests or personal relationships that could have appeared to influence the work reported in this paper.

## Appendix Supplementary materials

Image, application 1
