# Supplementary material for: Motion based ex vivo (MOTEX) culture of breast tumor slices sustains microenvironment composition
Source: Neoplasia. 2025 Aug 21;68:101221. doi: 10.1016/j.neo.2025.101221 (PMC12396401; doi:10.1016/j.neo.2025.101221)
Supplement: Supplementary file 1 [file mmc1.docx]

**Supplementary material**

***Supplementary Table 1:*** *Clinical characteristic of the resection material****.*** Overview of the primary breast cancer resection material samples, representing the *ex vivo* sensitivity to taxanes, receptor status, tumour histology and grade.

| Tumor code | Taxane sensitivity *ex vivo* | Receptor | Histology | Grade | Sample origin (study) |
| --- | --- | --- | --- | --- | --- |
| M382 | Sensitive | ER/PR+, HER2- | Ductal | 3 | Ladan, 2022 |
| M395 | Resistant | ER/PR+, HER2- | Ductal | 2 | Ladan, 2022 |
| M397 | unk. | ER+, PR/HER2- | unk. | unk. | *this study* |
| M412 | Sensitive | ER/PR+, HER2- | Lobular | 2 | Ladan, 2022 |
| M424 | Resistant | ER/PR+, HER2- | Lobular | 3 | Ladan, 2022 |
| M448 | Sensitive | ER/PR+, HER2- | Ductal | 2 | Ladan, 2022 |
| M457 | Resistant | unk. | unk. | unk. | *this study* |
| M459 | Sensitive | ER/PR+, HER2- | Lobular | 2 | Ladan, 2022 |
| M520 | Sensitive | ER/PR+, HER2- | unk. | unk. | *this study* |
| M532 | Sensitive | ER/PR+, HER2- | unk. | unk. | Komar, 2025 |
| M533 | unk. | ER/PR+, HER2- | Ductal | 2 | *this study* |
| M537 | unk. | unk. | unk. | unk. | *this study* |
| M538 | Sensitive | ER/PR+, HER2- | unk. | unk. | Komar, 2025 |
| M540 | unk. | ER/PR+, HER2- | Ductal | 3 | *this study* |
| M562 | unk. | unk. | unk. | unk. | *this study* |
| M566 | unk. | ER+, PR/HER2- | unk. | unk. | *this study* |
| M581 | Sensitive | ER/PR+, HER2- | unk. | unk. | Komar, 2025 |
| M584 | unk. | ER/PR+, HER2- | Ductal | 1 | *this study* |
| M587 | Sensitive | unk. | unk. | unk. | Komar, 2025 |
| M591 | Sensitive | ER+, PR/HER2- | Lobular | 1 | Komar, 2025 |
| M592 | unk. | ER/PR+, HER2- | Mucinous | 2 | *this study* |
| M611 | unk. | ER/PR+, HER2- | Ductal | 3 | *this study* |
| M636 | unk. | unk. | unk. | unk. | *this study* |

***Supplementary Table 2:*** *Clinical characteristic of the biopsy samples.* Overview of the primary breast cancer biopsy material samples, representing the *ex vivo* sensitivity to FAC and *in vivo* sensitivity to anthracycline-based chemotherapy and the receptor expression with the tumour grade.

| Tumor code | Sensitivity *ex vivo* | Sensitivity *in vivo* | ER | HER2 | | | Grade |
| --- | --- | --- | --- | --- | --- | --- | --- |
| B3 | Intermediate | Intermediate | Positive | | Negative | 2 | |
| B5 | Intermediate | Intermediate | Positive | | Negative | 2 | |
| B8 | Intermediate | Intermediate | Positive | | Positive | *3* | |
| B9 | Intermediate | Intermediate | Positive | | Positive | 3 | |
| B10 | Intermediate | Sensitive (rCR) | Positive | | Negative | 2 | |
| B12 | Sensitive | Intermediate | Positive | | Negative | 2 | |
| B16 | Intermediate | Intermediate | Positive | | Positive | 2 | |
| B17 | Sensitive | Intermediate | Negative | | Negative | 2 | |
| B18 | Intermediate | Intermediate | Positive | | Negative | 3 | |
| B20 | Intermediate | Intermediate | Positive | | Positive | 2 | |
| B21 | Intermediate | Intermediate | Negative | | Negative | 2 | |
| B22 | Intermediate | Intermediate | Positive | | Negative | 2 | |
| B23 | Sensitive | Sensitive (rCR) | Negative | | Negative | 2 | |
| B27 | Intermediate | Intermediate | Negative | | Negative | 3 | |
| B34 | Sensitive | Sensitive (rCR) | Negative | | Negative | 3 | |
| B35 | Intermediate | Sensitive (non-rCR) | Positive | | Negative | 2 | |
| B38 | Intermediate | Intermediate | Positive | | Negative | 2 | |
| B39 | Intermediate | Intermediate | Negative | | Negative | 2 | |
| B40 | Sensitive | Intermediate | Positive | | Negative | 2 | |
| B41 | Intermediate | Intermediate | Negative | | Negative | 3 | |


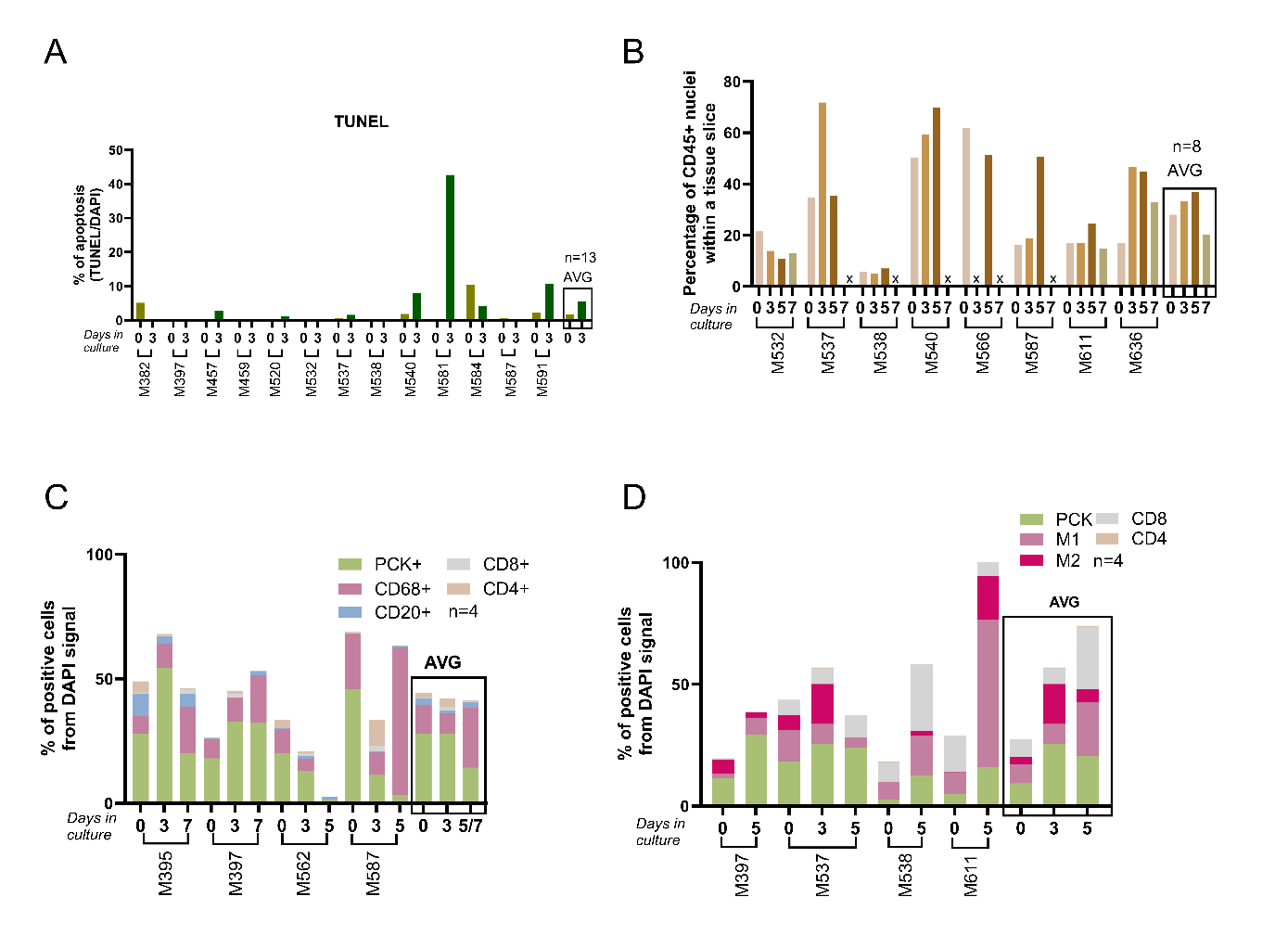


***Supplementary figure 1.*** *Survival of the TME in the MOTEX culture.* (A) Levels of apoptosis (TUNEL staining) before and after the MOTEX culture. Each bar represents an average TUNEL signal of one tissue slice (n=13). (B) Quantification of the CD45 levels after prolonged culture time of 3, 5 and 7 days (n=8). (C,D) Results of the Panel 1 MIF (C) and panel 2 MIF (D) for samples cultured for extended time (3, 5 and/or 7 days; n=4 per panel; n=8 total).


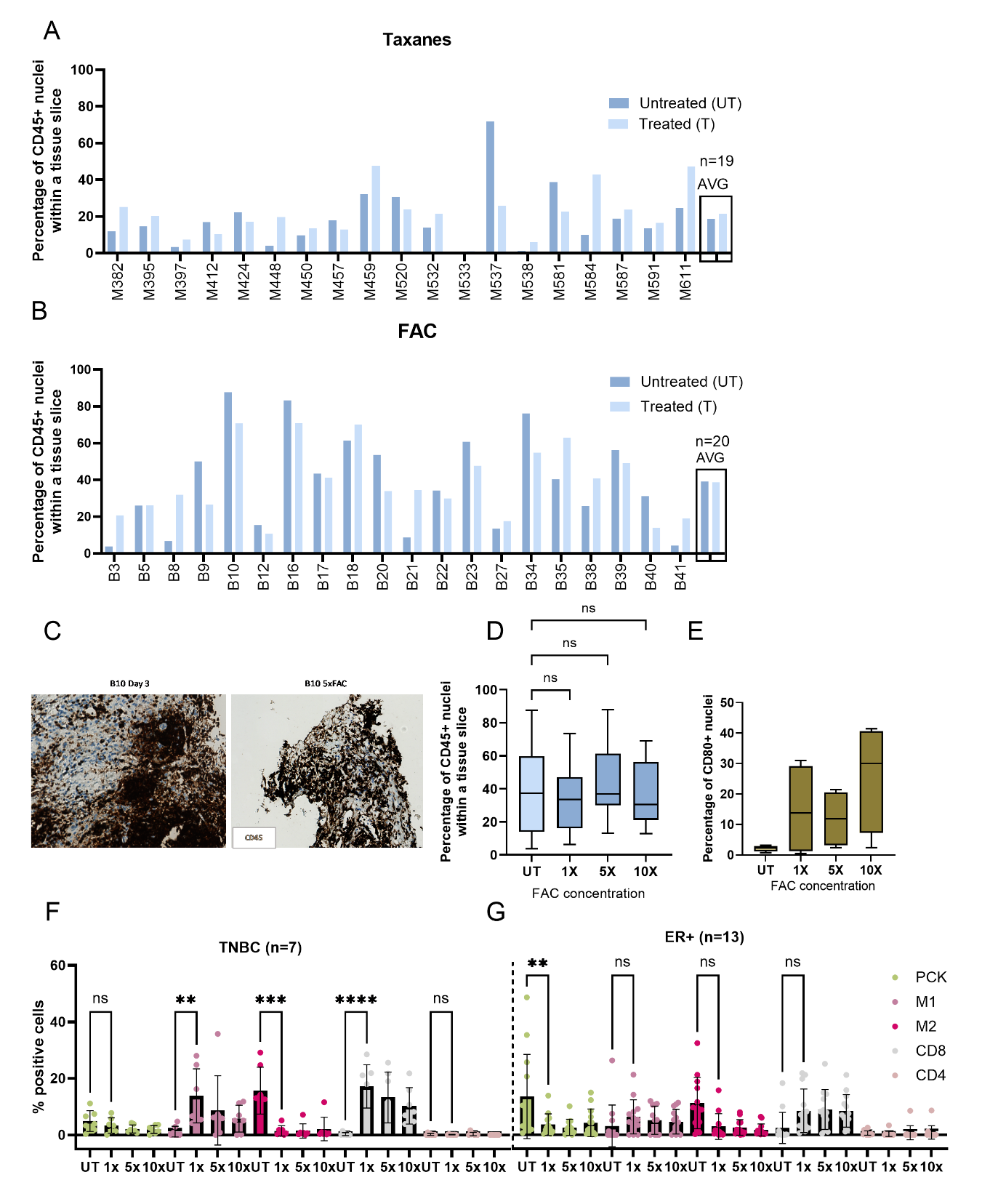


***Supplementary figure 2.*** *Effect of the treatment ex vivo on the TME.* (A) CD45 levels of BC resection material samples, represented for each individual samples separately, before and after treatment with taxanes *ex vivo* (n=19). (B) CD45 levels of BC biopsy material samples, represented for each individual samples separately, before and after treatment with FAC (average taken from all tested conditions) *ex vivo* (n=20). (C) Example image of a BC biopsy sample (B10), with a very high CD45 levels. (D) Representation of the differences in CD45 levels between the different concentrations of FAC. Significance was calculated by 2-way ANOVA test and indicated on the graph. (E) Quantification results of M1 macrophages (CD80+), after treatment with increasing FAC concentrations. Four samples were analysed here (n=4; B16, B27, B34 and B41), to confirm the specificity of the observed M2 macrophage decline presented in figure 3. (F,G) Representation of the results from Figure 3, with division of the biopsy samples between TNBC tumours (F) and ER-positive (G). Significant differences are indicated in the graph based on the 2-way ANOVA test.


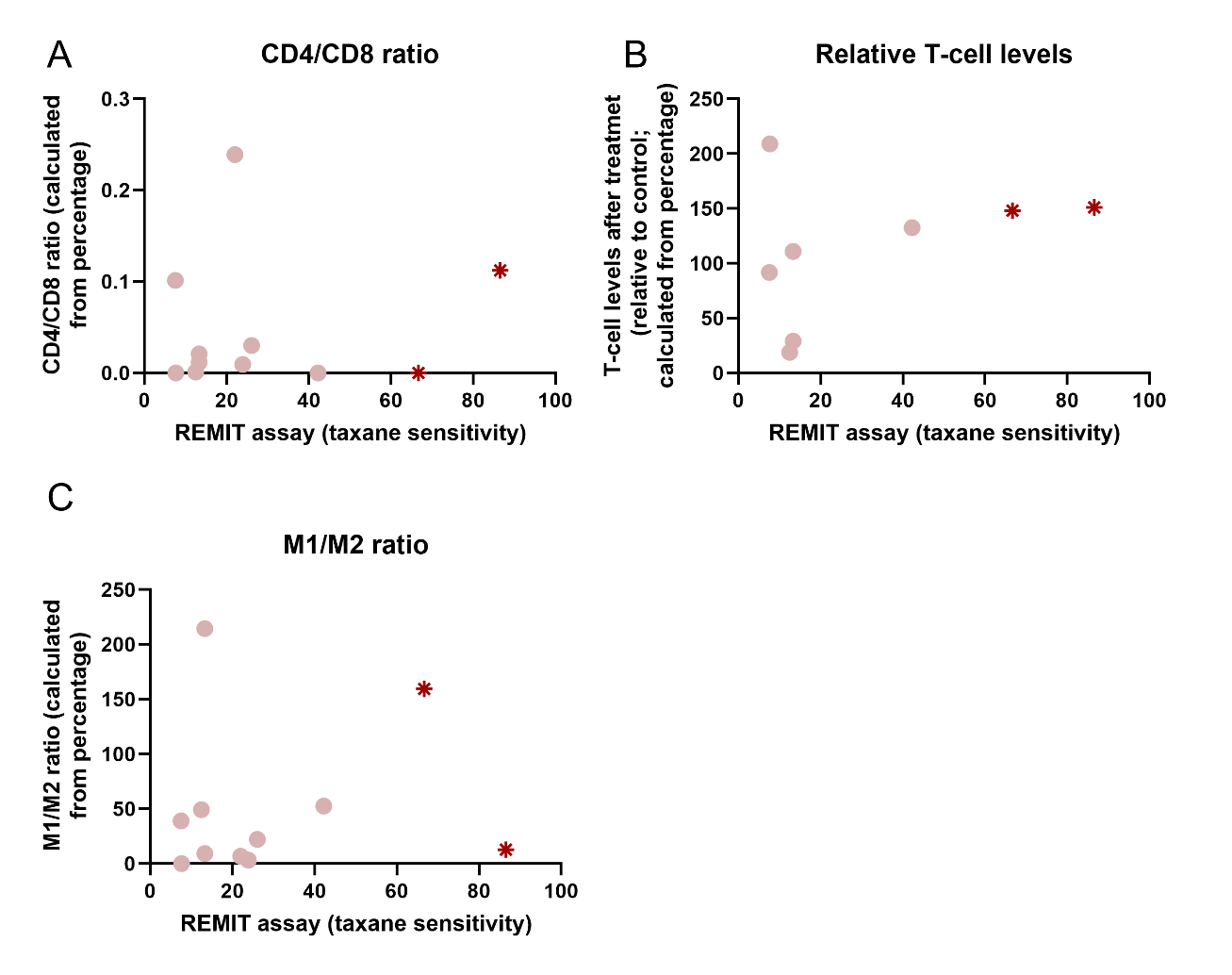


***Supplementary figure 3.*** *Assessment of the initial levels and composition of the TME and taxane response ex vivo.* Scatter plots representing the CD4⁺/CD8⁺ ratio (A), relative T-cell levels after treatment (the percentage of T-cell levels after treatment was calculated based on the initial T-cell levels) (B) and M1⁺/M2⁺ ratio (C) levels in relation to the taxane sensitivity. These results are based on the panel 2 MIF results represented in Figure 1 and 2. All graphs include the average levels calculated from day 0 and day 3 of untreated samples; exceptions are samples M412 and M587 were only day 3 was used and M457, M538, M581 and M591 were only day 0 was used (due to insufficient availability of material for the other time points). Panel 2 MIF results from sample M424 were not available.


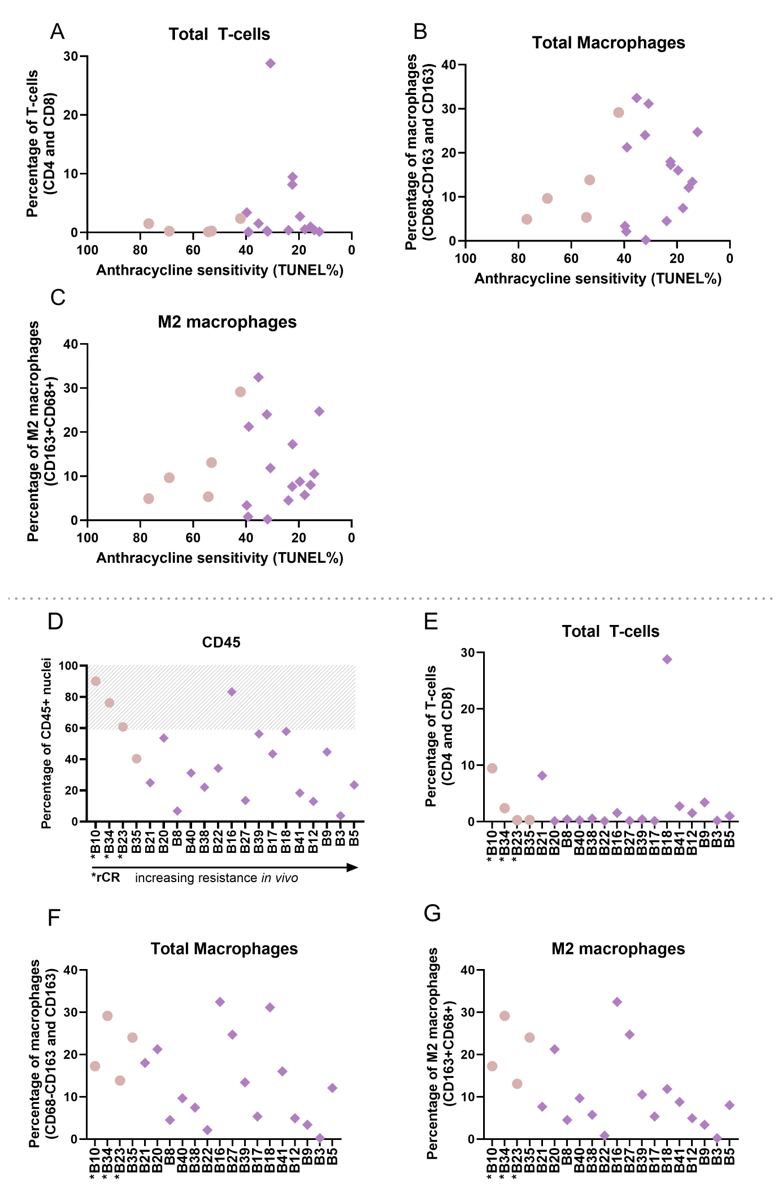


***Supplementary figure 4.*** *Assessment of the pre-treatment TME composition and sensitivity to FAC ex vivo and anthracycline-based chemotherapy in vivo.* (A-C) Scatter plots representing the total T-cell (A), total macrophages (B) and the M2⁺ macrophages (C) in relation to FAC sensitivity *ex vivo*. Scatter plot representing the CD45⁺ levels and thresholds applied to distinguish high and low infiltrated tumors. (E-G) Scatter plots representing the total T-cell (E), total macrophages (F) and the M2 macrophages (G) levels in relation to the increasing *in vivo* sensitivity to anthracycline-based treatment. Results from graphs A-C and E-G are based on the panel 2 MIF results represented in Figure 1 and 2. If available, the average levels calculated from day 0 and day 3 of untreated samples were included - samples B-5, 9, 10, 12, 18, 21, 38, 41) - with the exception of samples B-3, 8, 16, 17, 20, 22, 23, 27, 34, 35, 39 and 40, were only day 3 untreated samples was taken along.
